# Associations between the working experiences at frontline of COVID-19 pandemic and mental health of Korean public health doctors

**DOI:** 10.1186/s12888-021-03291-2

**Published:** 2021-06-09

**Authors:** Sangyoon Han, Sejin Choi, Seung Hyun Cho, Joonhyuk Lee, Je-Yeon Yun

**Affiliations:** 1 Eastern Seoul Detention Center, Ministry of Justice, Gwacheon-si, Republic of Korea; 2 Seoul Detention Center, Ministry of Justice, Gwacheon-si, Republic of Korea; 3 Seoul National University Hospital, Seoul, Republic of Korea; 4 Gaedo Public Health Center, Yeosu, Republic of Korea; 5 Hajang Public Health Center, Samcheok, Republic of Korea; 6 Yeongeon Student Support Center, Seoul National University College of Medicine, Seoul, Republic of Korea

**Keywords:** COVID-19, Health personnel, Mental health, Anxiety, Depression

## Abstract

**Background:**

Demographic, work environmental, and psychosocial features are associated with mental health of healthcare professionals at pandemic frontline. The current study aimed to find predictors of mental health for public health doctors from working experiences at frontline of COVID-19 pandemic.

**Methods:**

With first-come and first-served manner, 350 public health doctors with experiences of work at COVID-19 frontline participated online survey on August 2020. Mental health was defined using the total scores of the Patient Health Questionnaire-9, the Generalized Anxiety Disorder-7, the Perceived Stress Scale, and the Stanford Presenteeism Scale-6. Multivariate logistic regression models of mental health with lowest Akaike Information Criterion were determined among all combinations of working environments, perceived threats and satisfaction at frontline, and demographics that were significant (*P* < 0.05) in the univariate logistic regression.

**Results:**

Perceived distress, lowered self-efficacy at work, anxiety, and depressive mood were reported by 45.7, 34.6, 11.4, and 15.1% of respondents, respectively. Predictors of poor mental health found in the multivariate logistic regression analyses were environmental (insufficient personal protective equipment, workplace of screening center, prolonged workhours) and psychosocial (fear of infection and death, social stigma and rejection) aspects of working experiences at frontline. Satisfaction of monetary compensation and proactive coping (acceptance and willingness to volunteer at frontline) were predictive of better mental health.

**Conclusions:**

Sufficient supply of personal protective equipment and training on infection prevention at frontline, proper workhours and satisfactory monetary compensation, and psychological supports are required for better mental health of public health doctors at frontline of COVID-19 pandemic.

**Supplementary Information:**

The online version contains supplementary material available at 10.1186/s12888-021-03291-2.

## Background

Coronavirus disease 2019 (COVID-19) has become a worldwide pandemic since its appearance in December 2019 [1]. This new strain of coronavirus had an unknown virulence that seemed to cause high levels of fatality in Wuhan, China, the city where the infection of a human host was first recorded. The virus’ properties were still unknown when it began to spread worldwide and world authorities were alerted. The virus has caused a quarantine crisis unlike any seen before. Viral potency approaching that of the common cold made containing the disease an unforetold challenge for the authorities, while the unknown mortality, estimated as 0.1–25%, meant that COVID-19 required high levels of quarantine nonetheless [2]. The World Health Organization declared COVID-19 an international public health emergency on January 30, 2020 and by March 31st of 2021, more than 128,991,501 got infected, and more than 2,819,373 have died as a result of COVID-19 infection [3].

As of August 2020, the aggressive disease control measures proposed by the governments worldwide began to slow the spread of the disease. When an outbreak occurred, the Korean Centers for Disease Control and Prevention (KCDC) instantly moved public health doctors (PHDs) to areas where screening tests on all suspected COVID-19 patients could be performed, with the confirmed-positive patients quickly quarantined and provided necessary treatment [4]. The PHDs are a group of male doctors in South Korea who enlist for 3 years as an alternative to mandatory military duty. Approximately 700 doctors per year become a part of the PHD system, a government entity, and are assigned the task of providing healthcare to the medically marginalized population across the nation, especially in rural areas. For approximately 8 months, since February 2020, PHDs were the workforce of front-line disease control operations at screening centers, airport quarantine stations, makeshift shelters, and temporary isolation facilities, collecting swabs and managing patients. Some of them worked as epidemiologic investigators, doing contact tracing and triage of possible COVID-19 contacts. Of the many countries fighting against COVID-19, the Republic of Korea stood out by implementing expeditious countermeasures to the virus.

During the prolonged COVID-19 pandemic, however, physical exhaustion and psychological burnout of medical professionals at the frontline are increasing [5, 6]. In other words, reports of mental suffers among medical professionals at the COVID-19 frontline including perceived stress [7], anxiety [8–10], insomnia [11, 12], depressive mood [13], reduced self-efficiency in medical practice [14], traumatic or stress-related disorders [15], or suicidal ideation [16] have been increasing. First, in terms of the demographic features, younger-aged medical professionals in their earlier stage of career (with fewer years of work experience) and who provide direct care for the infected patients could be more vulnerable for the poor mental health outcome [10, 15, 17, 18]. Second, working environment factors of increased workload [19, 20], longer contact and higher exposure to patients [15, 21], insufficient supplies of protective equipment [22], risks of COVID-19 infection during medical practice [23], active duty at intensive care unit [24], lack of self-control over one’s daily routine [25], and needs of readjustment for upcoming situational changes [25] are related to the worse mental health of medical professionals. Of note, working in high-risk settings of closed wards treating COVID-19 patients, collecting respiratory specimen at screening center, or serving duty at emergency room during pandemic are associated with higher risk of poor mental health [10, 15, 17]. Third, for the psychosocial aspects, fear of the unknown and perceived threat of becoming infected [15, 18], perceived stigma and rejection from family members and neighborhood as a possible medium of propagating infection [8, 15, 18, 26], personal experience of quarantine after exposure to the COVID-positive patient [15], feelings of vulnerability and helplessness [15, 25] contribute to the perceived distress, feelings of isolation [18], and emotional reluctance to work [27]. Other risk population of poor mental health during pandemic are COVID-19 patients, quarantined persons, patients with pre-existing psychiatry disorder, and noninfectious chronic disease patients [12, 28].

Sustained suffer of poor mental health could lead to the reduced efficiency of medical practice and an intention to leave for healthcare professionals [29, 30]. However, to our knowledge, few studies investigated associated factors of mental health for drafted physicians in relation to the working experiences at frontline of COVID-19 pandemic. Therefore, the current study aimed to examine the mental health (= depressive mood, anxiety, perceived stress, and work-related self-efficacy) of PHDs drafted to the frontline of COVID-19 pandemic by way of the self-reporting questionnaires. Moreover, associations between working experience of PHDs at frontline versus mental health of PHDs were explored using the multivariate logistic regression analyses. We hypothesized that factors of working environments (such as workload [19, 20], working hours [15, 21], supplies of protective equipment [22], frequency of medical practice with more risks of infection [23], working location of dispatch [24], and capability of participating in the decision making [25]) and psychosocial aspects (such as perceived threat of becoming infected [15, 18], perceived stigma and rejection from others [8, 15, 18, 26], and feelings of vulnerability and helplessness [15, 25]) comprising the working experiences at frontline might be associated with mental health for PHD in Korea, who are younger-aged physicians in their earlier stage of career and serve direct care for the infected patients [10, 15, 17, 18].

## Methods

### Participants and data collection

From February 2020 to the present (as of April 2021), PHDs continue to be dispatched to the working locations of frontline such as rural public health centers, airport quarantine stations, and correctional facilities. At frontline of COVID-19 pandemic, PHDs conduct acquisition of respiratory specimen for diagnostic tests, epidemiological investigations to identify the paths of movements for confirmed-positive patients, and participation in patient care in the inpatient quarantine units, among others. The inclusion criteria were 1) Male doctors who had been serving active duty of PHD for Republic of Korea and had been members of Korean Association of Public Health Doctors as of August 2020 [population size = 1917], 2) Prior or current experiences of working at COVID-19 front-line between February and August of 2020 with duties of respiratory swab collection, epidemiologic investigation of the path of the confirmed patient for COVID-19, making triage (among the active monitoring, self-isolation, COVID-testing) of the people with possible recent contacts with confirmed individual, managing and treating COVID-positive patients, and 3) Those who willing to voluntarily participate in this web-based survey on August 2020. Cases who cannot satisfy all of these criteria were not allowed to participate the current study. The minimum number of necessary samples to satisfy the desired statistical constraints (confidence level = 95%; margin of error = 5%; population proportion = 50%; population size = 1917) calculated using the web-based ‘Sample Size Calculator’ program (https://www.calculator.net/math-calculator.html) was 321. Also taking into account the response rate of members of Korean Association of Public Health Doctors (≈ 18–19%), the final sample size was determined as 350 (out of the population size = 1,917).

**Fig. 1:**
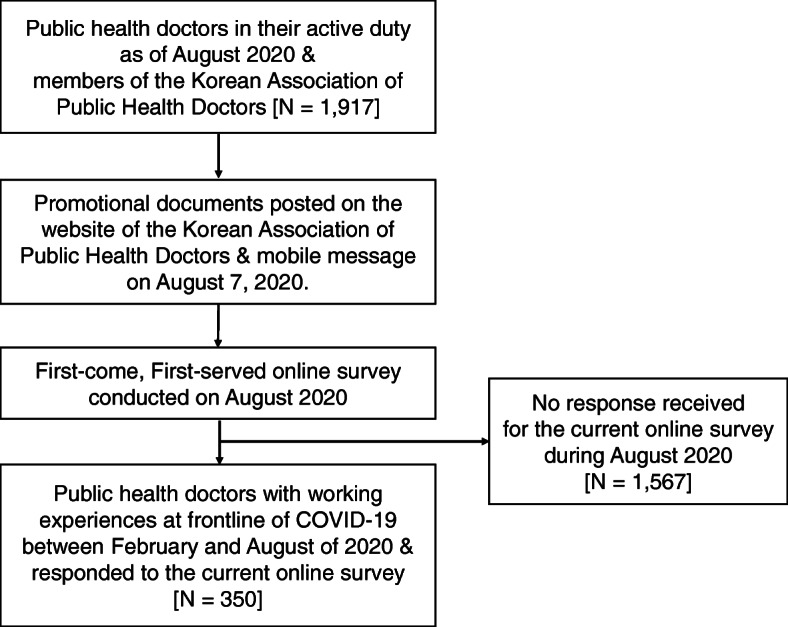
Flow chart of the study: recruitment & method of data collection

During this pandemic, it is suggested that data collection should be done off-site and on an online platform to prevent the further spread of COVID-19. Promotional documents for the current study was posted on the website of the Korean Association of Public Health Doctors (http://kaphd.org) and also delivered to the members of Korean Association of Public Health Doctors (*N* = 1917) by way of the mobile message on August 7, 2020. After reading the promotional document delivered, PHDs could click the web-based link for the online survey and could participate in the current study by responding to the questionnaires followed. Informed consent was indicated by clicking on the “start” button on the front page of the online survey. The survey was anonymous, and confidentiality of information was assured. Study participation was run by first-come, first-served manner for a total of 350 participants. In other words, after the 350 research participants have entered their answers into the web-based questionnaire, acquisition of further responses (or further study participation) by way of the web-based questionnaire was closed. Accordingly, this online survey was conducted from August 7 to August 18 of 2020 (as the cumulative number of study participants reached the target sample size of 350; Fig. 1). The current study was approved by the clinical research ethics committee of Seoul National University College of Medicine and Hospital (IRB No. 2007–152-1143). All methods were performed in accordance with relevant guidelines and regulations.

### Measurements

In the current study, items of the online survey (supplementary information: S1) were designed to explore possible associations between PHD mental health (depressive mood, anxiety, perceived stress, and presenteeism) and elements of their dispatch experience at the COVID-19 front line (working environment, workload, and perceived threat of COVID-19 infection). Most of all, assessments of mental health for PHDs was conducted using the four self-reports. First, depressive symptoms were measured using the Korean version of the Patient Health Questionnaire-9 (PHQ-9; S1. Part 5) [31–33]. Second, anxiety symptoms were measured by way of the Korean version of the Generalized Anxiety Disorder-7 scale (GAD-7; S1. Part 6) [34]. Third, perceived stress was explored using the Korean version of the Perceived Stress Scale (PSS; S1. Part 7) [35, 36]. Fourth, self-efficacy in one’s current PHD medical practice (work-related self-efficacy) was measured using the Stanford Presenteeism Scale-6 (SPS-6) [37] adapted for the COVID-19 pandemic (S1. Part 8).

Moreover, working experiences at frontline of COVID-19 and demographic factors of PHDs were also examined. First, basic demographic data including age, marital status, number of children, education level, and years of clinical experience were collected (S1. Part 1). Second, questions were asked about the working environment at the COVID-19 frontline, including timepoints/duration, urgency of dispatch, amount of training prior to dispatch, workload at the front line, and ease of acquiring protective equipment (S1. Parts 2 and 4). Third, PHDs were asked about the location and type of facility where they worked (S1. Part 2). Fourth, satisfaction with the experience of working at the frontline, such as subjective workload, perceived capability of participating in work-related decision making, satisfaction with the salary, and intention to volunteer for further dispatch, was examined using a 10-point Likert scale (S1. Part 4). Fourth, the perceived threat of COVID-19 infection was evaluated using 10 items, modified from Um et al. [38], a study that aimed to evaluate medical doctors’ perceptions of the risks associated with the 2017 Middle East Respiratory Syndrome (MERS) pandemic (S1. Part 3).

### Statistical analysis

To examine possible associations between mental health (depressive mood, anxiety, perceived stress, and work-related self-efficacy) and working experiences at frontline of COVID-19 pandemic (working environment and psychosocial aspects) for PHD using the logistic regressions, related variables were recoded into binary or categorical formats (Table 1). First, mental health of PHD (S1. Parts 5–8) were classified into 1) non-depressive (PHQ-9 total score < 10) vs. depressive (PHQ-9 total score ≥ 10) [31–33], 2) not anxious (GAD-7 total score < 10) vs. anxious (GAD-7 total score ≥ 10) [34], 3) not distressed (PSS total score < 18) vs. distressed (PSS total score ≥ 18) [35, 36], and 4) lower (SPS-6 total score < 19) vs. higher (SPS-6 total score ≥ 19) work-related self-efficacy [37]. Second, demographic (S1. Part 1) and working environmental (S1. Part 2 & items 4–1-1 to 4–1-4 of Part 4) variables were transformed into the categorical format (as written in Table 1). Third, Likert scale responses for the perceived threats of COVID-19 infection (S1. Part 3) were binary-transformed (never/not so much/unsure grouped as ‘NO’; possibly/certainly regarded as ‘YES’). Fourth, numerical rating responses for satisfaction of working experience at frontline (S1. Part 4) were recoded as YES (> 5) or NO (≤ 5).

**Table 1:** Demographic and Dispatch Experiences at COVID-19 Frontline [Working environment, Workload, Working Location, Satisfaction, and perceived threat of COVID-19 infection] (*N* = 350)

Item no.	Variables	Responses
Demographic and occupational characteristics		
S1.1–1	Age (mean (SD))	28.9 (2.11)
S1.1–3	Marriage (unmarried/married)	291 (83%)/59 (17%)
S1.1–4	Having children (no/yes)	330 (94%)/20 (6%)
S1.1–2	Level of education (college of medicine/medical school/master/doctor)	184 (53%)/145 (41%)/19 (5%)/2 (1%)
S1.1–6	Medical training prior to becoming PHD (general practitioner/intern/specialist)	220 (63%)/71 (20%)/59 (17%)
S1.1–7	Duration of experience for PHD duty (1st year/2nd year/3rd year)	146 (42%)/113 (32%)/91 (26%)
Working environment and workload of COVID-19 dispatch		
S1.2–1	Amount of education for COVID-19 frontline work prior to dispatch (< 8 h/≥ 8 h)	257 (73%)/93 (27%)
S1.2–2	Time interval between the notification and start of dispatch (< 7 days/≥ 7 days)	270 (77%)/80 (23%)
S1.4–1-1	Working of COVID-19 duties per week (< 35 h/35–45 h/> 45 h)	130 (37%)/132 (38%)/88 (25%)
S1.4–1-2	Number of nasopharyngeal swabs conducted per day (< 10/10–19/20–29/30–39/≥ 40)	82 (23%)/110 (31%)/71 (20%)/43 (12%)/44 (13%)
S1.4–1-4	Supply of protection equipment (always adequate-sometimes inadequate/often-always inadequate)	289 (83%)/61 (17%)
Working location of dispatch for COVID-19		
S1.2–3	Working at a COVID-19 triage center of your stationed city or county (yes/no)	303 (87%)/47 (13%)
S1.2–4	Dispatched outside of pre-stationed city (yes/no)	227 (65%)/123 (35%)
S1.2–6	Dispatched to Daegu (major outbreak during 2020/02–03) for COVID-19 management (yes/no)	123 (35%)/227 (65%)
S1.2–7	Form of COVID-19 frontline dispatched: screening center (yes/no)	298 (85%)/52 (15%)
	Form of COVID-19 frontline dispatched: airport/port quarantines (yes/no)	48 (14%)/302 (86%)
	Form of COVID-19 frontline dispatched: makeshift shelters (yes/no)	73 (21%)/277 (79%)
	Form of COVID-19 frontline dispatched: isolation facility for foreign entrants (yes/no)	66 (19%)/284 (81%)
	Form of COVID-19 frontline dispatched: intensive care unit of general hospital (yes/no)	73 (21%)/277 (79%)
Satisfaction for experience of working at COVID-19 frontline [0(not at all)-5(average)-10(absolutely yes)]		
S1.4–2	Subjective workload (> 5 (more than average)/≤ 5 (average or less))	198 (57%)/152 (43%)
S1.4–3	Perceived capability of participating in the decision making (> 5)/≤ 5)	112 (32%)/238 (68%)
S1.4–4	Satisfaction for the monetary compensation (> 5 (more than average)/≤ 5 (average or less))	90 (26%)/260 (74%)
S1.4–5	Willingness to further volunteering for dispatch (> 5 (more than average)/≤ 5 (average or less))	108 (31%)/242 (69%)
S1.4–6	Experience of consultation with a psychiatrist during or after the dispatch (yes/no)	7 (2%)/343 (98%)
Perceived threat of COVID-19 infection [never/not so much/unsure/possibly/certainly]		
S1.3–1	COVID-19 duty puts me at great risk (possibly-certain/never-unsure)	209 (60%)/141 (40%)
S1.3–2	I feel more stress during COVID-19 duty than other tasks (possibly-certain/never-unsure)	264 (75%)/86 (25%)
S1.3–3	I can accept the risk of caring for COVID-19 patients. (unsure-certain/never-not so much)	296 (85%)/54 (15%)
S1.3–4	I am afraid of falling ill with COVID-19 (possibly-certain/never-unsure)	289 (83%)/61 (17%)
S1.3–5	I have little control over whether I get infected or not. (possibly-certain/never-unsure)	135 (39%)/215 (61%)
S1.3–6	I have little chance of survival if I were to get COVID-19 (possibly-certain/never-unsure)	8 (2%)/342 (98%)
S1.3–7	If possible, I want to resign from COVID-19 duty (possibly-certain/never-unsure)	204 (58%)/146 (42%)
S1.3–8	I am afraid I will pass COVID-19 to others. (possibly-certain/never-unsure)	301 (86%)/49 (14%)
S1.3–9	My family & friends are worried they might get infected thru me (possibly-certain/never-unsure)	234 (67%)/116 (33%)
S1.3–10	People avoid me because of my COVID-19 duty (possibly-certain/never-unsure)	85 (24%)/265 (76%)

Using the univariate binary logistic regression analyses, demographics or variables of working experiences at frontline that significantly explained mental health [non-depressive vs. depressive, non-anxious vs. anxious, non-distressed vs. distressed, and lower vs. higher work-related self-efficacy] were retrieved (all Ps < 0.05; Table 2). Finally, multivariate logistic regression analyses using an R package of *glmulti* [with options of: level = 1 (no interaction considered); method = “h” (exhaustive search); crit = “aic” (Akaike information criterion as criteria of model selection); fitfunction = “glm” (use *glm* function for regression analyses)] found best model of explaining mental health among all possible combinations of working experiences and demographics that were significant (*P* < 0.05) in the univariate binary logistic regression (Table 3; refer to S2. R code for multivariate logistic regression in the supplementary information). Data were analyzed using R version 4.0.2 (R Core Development Team, 2020).

**Table 2:** Univariate Associations between the Dispatch Experiences at COVID-19 Frontline versus Mental Health status of PHD

Item number	Variables		PHQ-9 (depressive mood)				GAD-7 (Anxiety)				PSS (perceived stress)				SPS-6 (work-related self-efficacy)			
			Normal or Mild (***N*** = 297)	Moderate to Severe (***N*** = 53)	OR (univariate)	*P* value	Normal or Mild (***N*** = 310)	Moderate to Severe (***N*** = 40)	OR (univariable)	*P* value	Low (***N*** = 190)	High (***N*** = 160)	OR (univariate)	*P* value	Low (***N*** = 229)	High (***N*** = 121)	OR (univariate)	*P* value
**Demographic and occupational characteristics**																		
Q.1-2	Level of education	College of medicine	156 (84.8)	28 (15.2)	1 [reference]		163 (88.6)	21 (11.4)	1 [reference]		103 (56.0)	81 (44.0)	1 [reference]		128 (69.6)	56 (30.4)	1 [reference]	
		Medical school	122 (84.1)	23 (15.9)	1.05 (0.57-1.91)	0.873	128 (88.3)	17 (11.7)	1.03 (0.52-2.03)	0.93	73 (50.3)	72 (49.7)	1.25 (0.81-1.94)	0.309	91 (62.8)	54 (37.2)	1.36 (0.86-2.15)	0.194
		Master's degree	17 (89.5)	2 (10.5)	0.66 (0.10-2.46)	0.586	17 (89.5)	2 (10.5)	0.91 (0.14-3.50)	0.908	13 (68.4)	6 (31.6)	0.59 (0.20-1.55)	0.301	10 (52.6)	9 (47.4)	2.06 (0.78-5.38)	0.138
		Doctoral degree	2 (100.0)	0 (0.0)	0.00 (NA)	0.989	2 (100.0)	0 (0.0)	0.00 (NA)	0.99	1 (50.0)	1 (50.0)	1.27 (0.05-32.48)	0.866	0 (0.0)	2 (100.0)	inf (NA)	0.98
Q.1-3	Marriage	No	246 (84.5)	45 (15.5)	1 [reference]		257 (88.3)	34 (11.7)	1 [reference]		160 (55.0)	131 (45.0)	1 [reference]		197 (67.7)	94 (32.3)	1 [reference]	
		Yes	51 (86.4)	8 (13.6)	0.86 (0.36-1.84)	0.71	53 (89.8)	6 (10.2)	0.86 (0.31-2.01)	0.739	30 (50.8)	29 (49.2)	1.18 (0.67-2.07)	0.561	32 (54.2)	27 (45.8)	**1.77 (1.00-3.12)**	0.049*
Q.1-4	Children	No	280 (84.8)	50 (15.2)	1 [reference]		292 (88.5)	38 (11.5)	1 [reference]		180 (54.5)	150 (45.5)	1 [reference]		220 (66.7)	110 (33.3)	1 [reference]	
		Yes	17 (85.0)	3 (15.0)	0.99 (0.22-3.08)	0.985	18 (90.0)	2 (10.0)	0.85 (0.13-3.12)	0.836	10 (50.0)	10 (50.0)	1.20 (0.48-3.00)	0.692	9 (45.0)	11 (55.0)	2.44 (0.98-6.23)	0.054
Q.1-6	Training	General physician	179 (81.4)	41 (18.6)	1 [reference]		189 (85.9)	31 (14.1)	1 [reference]		107 (48.6)	113 (51.4)	1 [reference]		143 (65.0)	77 (35.0)	1 [reference]	
		Finished Intern year	64 (90.1)	7 (9.9)	0.48 (0.19-1.06)	0.089	66 (93.0)	5 (7.0)	0.46 (0.15-1.14)	0.124	45 (63.4)	26 (36.6)	**0.55 (0.31-0.94)**	0.032*	50 (70.4)	21 (29.6)	0.78 (0.43-1.38)	0.401
		Specialist	54 (91.5)	5 (8.5)	0.40 (0.13-0.99)	0.069	55 (93.2)	4 (6.8)	0.44 (0.13-1.18)	0.141	38 (64.4)	21 (35.6)	**0.52 (0.28-0.94)**	0.033*	36 (61.0)	23 (39.0)	1.19 (0.65-2.13)	0.571
Q.1-7	PHD year	First year	127 (87.0)	19 (13.0)	1 [reference]		135 (92.5)	11 (7.5)	1 [reference]		92 (63.0)	54 (37.0)	1 [reference]		102 (69.9)	44 (30.1)	1 [reference]	
		Second year	95 (84.1)	18 (15.9)	1.27 (0.63-2.55)	0.507	99 (87.6)	14 (12.4)	1.74 (0.76-4.07)	0.194	53 (46.9)	60 (53.1)	**1.93 (1.17-3.19)**	0.01*	68 (60.2)	45 (39.8)	1.53 (0.92-2.58)	0.104
		Third year	75 (82.4)	16 (17.6)	1.43 (0.68-2.94)	0.337	76 (83.5)	15 (16.5)	**2.42 (1.07-5.67)**	0.036*	45 (49.5)	46 (50.5)	**1.74 (1.03-2.97)**	0.041*	59 (64.8)	32 (35.2)	1.26 (0.72-2.19)	0.42
**Working environment in relation to the COVID-19 dispatch**																		
Q.2-1	Prior Education	< 8 hours	216 (84.0)	41 (16.0)	1 [reference]		224 (87.2)	33 (12.8)	1 [reference]		130 (50.6)	127 (49.4)	1 [reference]		163 (63.4)	94 (36.6)	1 [reference]	
		>= 8 hours	81 (87.1)	12 (12.9)	0.78 (0.38-1.52)	0.483	86 (92.5)	7 (7.5)	0.55 (0.22-1.23)	0.173	60 (64.5)	33 (35.5)	**0.56 (0.34-0.91)**	0.022*	66 (71.0)	27 (29.0)	0.71 (0.42-1.18)	0.191
Q.2-2	Notice	< 1 week	98 (79.7)	25 (20.3)	1 [reference]		100 (81.3)	23 (18.7)	1 [reference]		55 (44.7)	68 (55.3)	1 [reference]		72 (58.5)	51 (41.5)	1 [reference]	
		>= 1week	199 (87.7)	28 (12.3)	**0.55 (0.31-1.00)**	0.048*	210 (92.5)	17 (7.5)	**0.35 (0.18-0.68)**	0.002*	135 (59.5)	92 (40.5)	**0.55 (0.35-0.86)**	0.008*	157 (69.2)	70 (30.8)	**0.63 (0.40-0.99)**	0.047*
Q.4-1-1	Working hour	< 35 hours/week	118 (90.8)	12 (9.2)	1 [reference]		120 (92.3)	10 (7.7)	1 [reference]		75 (57.7)	55 (42.3)	1 [reference]		82 (63.1)	48 (36.9)	1 [reference]	
		35-45 hours/week	110 (83.3)	22 (16.7)	1.97 (0.94-4.28)	0.077	114 (86.4)	18 (13.6)	1.89 (0.85-4.43)	0.124	71 (53.8)	61 (46.2)	1.17 (0.72-1.91)	0.525	84 (63.6)	48 (36.4)	0.98 (0.59-1.61)	0.925
		> 45 hours/week	69 (78.4)	19 (21.6)	**2.71 (1.25-6.06)**	0.012*	76 (86.4)	12 (13.6)	1.89 (0.78-4.70)	0.158	44 (50.0)	44 (50.0)	1.36 (0.79-2.35)	0.264	63 (71.6)	25 (28.4)	0.68 (0.37-1.21)	0.192
Q.4-1-2	Nuber of swabs per day	<10	74 (90.2)	8 (9.8)	1 [reference]		75 (91.5)	7 (8.5)	1 [reference]		48 (58.5)	34 (41.5)	1 [reference]		51 (62.2)	31 (37.8)	1 [reference]	
		>=10 & <20	94 (85.5)	16 (14.5)	1.57 (0.65-4.07)	0.324	99 (90.0)	11 (10.0)	1.19 (0.45-3.37)	0.731	60 (54.5)	50 (45.5)	1.18 (0.66-2.10)	0.581	74 (67.3)	36 (32.7)	0.80 (0.44-1.46)	0.466
		>=20 & <30	57 (80.3)	14 (19.7)	2.27 (0.91-6.04)	0.085	59 (83.1)	12 (16.9)	2.18 (0.82-6.18)	0.124	36 (50.7)	35 (49.3)	1.37 (0.72-2.61)	0.332	43 (60.6)	28 (39.4)	1.07 (0.56-2.06)	0.836
		>=30 & <40	36 (83.7)	7 (16.3)	1.80 (0.59-5.40)	0.291	39 (90.7)	4 (9.3)	1.10 (0.27-3.87)	0.886	23 (53.5)	20 (46.5)	1.23 (0.58-2.59)	0.589	35 (81.4)	8 (18.6)	**0.38 (0.15-0.88)**	0.031*
		>=40	36 (81.8)	8 (18.2)	2.06 (0.70-6.02)	0.182	38 (86.4)	6 (13.6)	1.69 (0.51-5.44)	0.374	23 (52.3)	21 (47.7)	1.29 (0.62-2.70)	0.5	26 (59.1)	18 (40.9)	1.14 (0.53-2.40)	0.733
Q.4-1-4	PPE	insufficient	39 (63.9)	22 (36.1)	1 [reference]		43 (70.5)	18 (29.5)	1 [reference]		18 (29.5)	43 (70.5)	1 [reference]		34 (55.7)	27 (44.3)	1 [reference]	
		sufficient	258 (89.3)	31 (10.7)	**0.21 (0.11-0.41)**	<0.001*	267 (92.4)	22 (7.6)	**0.20 (0.10-0.40)**	<0.001*	172 (59.5)	117 (40.5)	**0.28 (0.15-0.51)**	<0.001*	195 (67.5)	94 (32.5)	0.61 (0.35-1.07)	0.082
**Working location of dispatch for COVID-19**																		
Q. 2-3	Worked at own area	No	44 (93.6)	3 (6.4)	1 [reference]		44 (93.6)	3 (6.4)	1 [reference]		34 (72.3)	13 (27.7)	1 [reference]		28 (59.6)	19 (40.4)	1 [reference]	
		Yes	253 (83.5)	50 (16.5)	2.90 (1.01-12.27)	0.084	266 (87.8)	37 (12.2)	2.04 (0.70-8.70)	0.252	156 (51.5)	147 (48.5)	**2.46 (1.28-5.01)**	0.009*	201 (66.3)	102 (33.7)	0.75 (0.40-1.42)	0.366
Q.2-4	Dispatched	No	103 (83.7)	20 (16.3)	1 [reference]		110 (89.4)	13 (10.6)	1 [reference]		71 (57.7)	52 (42.3)	1 [reference]		80 (65.0)	43 (35.0)	1 [reference]	
		Yes	194 (85.5)	33 (14.5)	0.88 (0.48-1.63)	0.668	200 (88.1)	27 (11.9)	1.14 (0.58-2.37)	0.71	119 (52.4)	108 (47.6)	1.24 (0.80-1.93)	0.342	149 (65.6)	78 (34.4)	0.97 (0.62-1.55)	0.911
Q.2-7	Dispatch to epicenter	No	191 (84.1)	36 (15.9)	1 [reference]		202 (89.0)	25 (11.0)	1 [reference]		121 (53.3)	106 (46.7)	1 [reference]		151 (66.5)	76 (33.5)	1 [reference]	
		Yes	106 (86.2)	17 (13.8)	0.85 (0.45-1.57)	0.612	108 (87.8)	15 (12.2)	1.12 (0.56-2.20)	0.74	69 (56.1)	54 (43.9)	0.89 (0.57-1.39)	0.617	78 (63.4)	45 (36.6)	1.15 (0.72-1.81)	0.56
	Screening center	No	50 (96.2)	2 (3.8)	1 [reference]		50 (96.2)	2 (3.8)	1 [reference]		41 (78.8)	11 (21.2)	1 [reference]		33 (63.5)	19 (36.5)	1 [reference]	
		Yes	247 (82.9)	51 (17.1)	**5.16 (1.53-32.19)**	0.026*	260 (87.2)	38 (12.8)	3.65 (1.07-22.91)	0.081	149 (50.0)	149 (50.0)	**3.73 (1.91-7.88)**	<0.001*	196 (65.8)	102 (34.2)	0.90 (0.49-1.69)	0.747
	Airport qurantine	No	255 (84.4)	47 (15.6)	1 [reference]		264 (87.4)	38 (12.6)	1 [reference]		164 (54.3)	138 (45.7)	1 [reference]		204 (67.5)	98 (32.5)	1 [reference]	
		Yes	42 (87.5)	6 (12.5)	0.78 (0.28-1.80)	0.583	46 (95.8)	2 (4.2)	0.30 (0.05-1.03)	0.107	26 (54.2)	22 (45.8)	1.01 (0.54-1.85)	0.986	25 (52.1)	23 (47.9)	**1.92 (1.03-3.55)**	0.039*
	Makeshift shelters	No	232 (83.8)	45 (16.2)	1 [reference]		244 (88.1)	33 (11.9)	1 [reference]		149 (53.8)	128 (46.2)	1 [reference]		176 (63.5)	101 (36.5)	1 [reference]	
		Yes	65 (89.0)	8 (11.0)	0.63 (0.27-1.35)	0.266	66 (90.4)	7 (9.6)	0.78 (0.31-1.76)	0.579	41 (56.2)	32 (43.8)	0.91 (0.54-1.52)	0.717	53 (72.6)	20 (27.4)	0.66 (0.37-1.15)	0.149
	Isolation facility	No	240 (84.5)	44 (15.5)	1 [reference]		249 (87.7)	35 (12.3)	1 [reference]		152 (53.5)	132 (46.5)	1 [reference]		183 (64.4)	101 (35.6)	1 [reference]	
		Yes	57 (86.4)	9 (13.6)	0.86 (0.38-1.79)	0.705	61 (92.4)	5 (7.6)	0.58 (0.19-1.43)	0.28	38 (57.6)	28 (42.4)	0.85 (0.49-1.45)	0.552	46 (69.7)	20 (30.3)	0.79 (0.43-1.39)	0.419
	Hospital ICU	No	232 (83.8)	45 (16.2)	1 [reference]		244 (88.1)	33 (11.9)	1 [reference]		144 (52.0)	133 (48.0)	1 [reference]		179 (64.6)	98 (35.4)	1 [reference]	
		Yes	65 (89.0)	8 (11.0)	0.63 (0.27-1.35)	0.266	66 (90.4)	7 (9.6)	0.78 (0.31-1.76)	0.579	46 (63.0)	27 (37.0)	0.64 (0.37-1.07)	0.094	50 (68.5)	23 (31.5)	0.84 (0.48-1.45)	0.536
**Satisfaction for experience of working at COVID-19 frontline**																		
Q.4-2	Subjective workload	Not Tough	141 (92.8)	11 (7.2)	1 [reference]		145 (95.4)	7 (4.6)	1 [reference]		98 (64.5)	54 (35.5)	1 [reference]		108 (71.1)	44 (28.9)	1 [reference]	
		Tough	156 (78.8)	42 (21.2)	**3.45 (1.77-7.28)**	0.001*	165 (83.3)	33 (16.7)	**4.14 (1.88-10.46)**	0.001*	92 (46.5)	106 (53.5)	**2.09 (1.36-3.24)**	<0.001*	121 (61.1)	77 (38.9)	1.56 (1.00-2.47)	0.053
Q.4-3	Capability of participating in the decision making	No	195 (81.9)	43 (18.1)	1 [reference]		204 (85.7)	34 (14.3)	1 [reference]		117 (49.2)	121 (50.8)	1 [reference]		147 (61.8)	91 (38.2)	1 [reference]	
		Yes	102 (91.1)	10 (8.9)	**0.44 (0.20-0.89)**	0.029*	106 (94.6)	6 (5.4)	**0.34 (0.13-0.78)**	0.019*	73 (65.2)	39 (34.8)	**0.52 (0.32-0.82)**	0.005*	82 (73.2)	30 (26.8)	**0.59 (0.36-0.96)**	0.037*
Q.4-4	Satisfaction for the monetary compensation	No	216 (83.1)	44 (16.9)	1 [reference]		226 (86.9)	34 (13.1)	1 [reference]		125 (48.1)	135 (51.9)	1 [reference]		165 (63.5)	95 (36.5)	1 [reference]	
		Yes	81 (90.0)	9 (10.0)	0.55 (0.24-1.12)	0.119	84 (93.3)	6 (6.7)	0.47 (0.17-1.10)	0.106	65 (72.2)	25 (27.8)	**0.36 (0.21-0.59)**	<0.001*	64 (71.1)	26 (28.9)	0.71 (0.41-1.18)	0.19
Q.4-5	Willingness to further volunteering for dispatch	No	197 (81.4)	45 (18.6)	1 [reference]		207 (85.5)	35 (14.5)	1 [reference]		114 (47.1)	128 (52.9)	1 [reference]		142 (58.7)	100 (41.3)	1 [reference]	
		Yes	100 (92.6)	8 (7.4)	**0.35 (0.15-0.73)**	0.009*	103 (95.4)	5 (4.6)	**0.29 (0.10-0.69)**	0.011*	76 (70.4)	32 (29.6)	**0.38 (0.23-0.60)**	<0.001*	87 (80.6)	21 (19.4)	**0.34 (0.20-0.58)**	<0.001*
Q.4-6	Consulted with psychiatrist	No	293 (85.4)	50 (14.6)	1 [reference]		305 (88.9)	38 (11.1)	1 [reference]		187 (54.5)	156 (45.5)	1 [reference]		224 (65.3)	119 (34.7)	1 [reference]	
		Yes	4 (57.1)	3 (42.9)	4.39 (0.84-20.51)	0.057	5 (71.4)	2 (28.6)	3.21 (0.45-15.47)	0.172	3 (42.9)	4 (57.1)	1.60 (0.35-8.22)	0.543	5 (71.4)	2 (28.6)	0.75 (0.11-3.55)	0.737
**Perceived threat of COVID-19 infection**																		
Q.3-1	COVID-19 duty puts me at great risk	No	134 (95.0)	7 (5.0)	1 [reference]		137 (97.2)	4 (2.8)	1 [reference]		98 (69.5)	43 (30.5)	1 [reference]		110 (78.0)	31 (22.0)	1 [reference]	
		Yes	163 (78.0)	46 (22.0)	**5.40 (2.51-13.45)**	<0.001*	173 (82.8)	36 (17.2)	**7.13 (2.77-24.25)**	<0.001*	92 (44.0)	117 (56.0)	**2.90 (1.86-4.58)**	<0.001*	119 (56.9)	90 (43.1)	**2.68 (1.67-4.40)**	<0.001*
Q.3-2	I feel more stress for COVID-19 duty than other tasks	No	84 (97.7)	2 (2.3)	1 [reference]		86 (100.0)	0 (0.0)	1 [reference]		68 (79.1)	18 (20.9)	1 [reference]		76 (88.4)	10 (11.6)	1 [reference]	
		Yes	213 (80.7)	51 (19.3)	**10.06 (3.03-62.33)**	0.002*	224 (84.8)	40 (15.2)	inf(NA)	0.988	122 (46.2)	142 (53.8)	**4.40 (2.53-7.99)**	<0.001*	153 (58.0)	111 (42.0)	**5.51 (2.85-11.78)**	<0.001*
Q.3-3	I can accept the risk of caring for COVID-19 patients	No	36 (66.7)	18 (33.3)	1 [reference]		43 (79.6)	11 (20.4)	1 [reference]		24 (44.4)	30 (55.6)	1 [reference]		29 (53.7)	25 (46.3)	1 [reference]	
		Yes	261 (88.2)	35 (11.8)	**0.27 (0.14-0.53)**	<0.001*	267 (90.2)	29 (9.8)	**0.42 (0.20-0.94)**	0.028*	166 (56.1)	130 (43.9)	0.63 (0.35-1.12)	0.116	200 (67.6)	96 (32.4)	0.56 (0.31-1.01)	0.051
Q.3-4	I am afraid of falling ill with COVID-19	No	51 (83.6)	10 (16.4)	1 [reference]		56 (91.8)	5 (8.2)	1 [reference]		36 (59.0)	25 (41.0)	1 [reference]		45 (73.8)	16 (26.2)	1 [reference]	
		Yes	246 (85.1)	43 (14.9)	0.89 (0.43-1.98)	0.764	254 (87.9)	35 (12.1)	1.54 (0.63-4.65)	0.386	154 (53.3)	135 (46.7)	1.26 (0.72-2.23)	0.415	184 (63.7)	105 (36.3)	1.60 (0.88-3.06)	0.134
Q.3-5	I have little control over whether I get infected or not	No	192 (89.3)	23 (10.7)	1 [reference]		199 (92.6)	16 (7.4)	1 [reference]		130 (60.5)	85 (39.5)	1 [reference]		150 (69.8)	65 (30.2)	1 [reference]	
		Yes	105 (77.8)	30 (22.2)	**2.39 (1.32-4.35)**	0.004*	111 (82.2)	24 (17.8)	**2.69 (1.38-5.36)**	0.004*	60 (44.4)	75 (55.6)	**1.91 (1.24-2.96)**	0.004*	79 (58.5)	56 (41.5)	**1.64 (1.04-2.57)**	0.032*
Q.3-6	I have little chance of survival if I were to get COVID-19	No	293 (85.7)	49 (14.3)	1 [reference]		306 (89.5)	36 (10.5)	1 [reference]		187 (54.7)	155 (45.3)	1 [reference]		226 (66.1)	116 (33.9)	1 [reference]	
		Yes	4 (50.0)	4 (50.0)	**5.98 (1.37-26.05)**	0.013*	4 (50.0)	4 (50.0)	**8.50 (1.94-37.37)**	0.003*	3 (37.5)	5 (62.5)	2.01 (0.49-9.93)	0.344	3 (37.5)	5 (62.5)	3.25 (0.78-16.05)	0.111
Q.3-7	If it were possible, I want to resign from COVID-19 duty	No	135 (92.5)	11 (7.5)	1 [reference]		138 (94.5)	8 (5.5)	1 [reference]		106 (72.6)	40 (27.4)	1 [reference]		109 (74.7)	37 (25.3)	1 [reference]	
		Yes	162 (79.4)	42 (20.6)	**3.18 (1.63-6.72)**	0.001*	172 (84.3)	32 (15.7)	**3.21 (1.50-7.69)**	0.005*	84 (41.2)	120 (58.8)	**3.79 (2.41-6.03)**	<0.001*	120 (58.8)	84 (41.2)	**2.06 (1.30-3.31)**	0.002*
Q.3-8	I am afraid I will pass COVID-19 to others	No	45 (91.8)	4 (8.2)	1 [reference]		48 (98.0)	1 (2.0)	1 [reference]		33 (67.3)	16 (32.7)	1 [reference]		34 (69.4)	15 (30.6)	1 [reference]	
		Yes	252 (83.7)	49 (16.3)	2.19 (0.84-7.50)	0.151	262 (87.0)	39 (13.0)	7.15 (1.49-128.28)	0.055	157 (52.2)	144 (47.8)	1.89 (1.01-3.66)	0.05	195 (64.8)	106 (35.2)	1.23 (0.65-2.42)	0.53
Q.3-9	Family & friends are worried they might get infected thru me	No	107 (92.2)	9 (7.8)	1 [reference]		113 (97.4)	3 (2.6)	1 [reference]		75 (64.7)	41 (35.3)	1 [reference]		83 (71.6)	33 (28.4)	1 [reference]	
		Yes	190 (81.2)	44 (18.8)	**2.75 (1.35-6.22)**	0.009*	197 (84.2)	37 (15.8)	**7.07 (2.48-29.77)**	0.001*	115 (49.1)	119 (50.9)	**1.89 (1.20-3.01)**	0.006*	146 (62.4)	88 (37.6)	1.52 (0.94-2.48)	0.091
Q.3-10	People avoid me because of my COVID-19 duty	No	237 (89.4)	28 (10.6)	1 [reference]		242 (91.3)	23 (8.7)	1 [reference]		153 (57.7)	112 (42.3)	1 [reference]		180 (67.9)	85 (32.1)	1 [reference]	
		Yes	60 (70.6)	25 (29.4)	**3.53 (1.91-6.50)**	<0.001*	68 (80.0)	17 (20.0)	**2.63 (1.31-5.19)**	0.005*	37 (43.5)	48 (56.5)	**1.77 (1.08-2.92)**	0.023*	49 (57.6)	36 (42.4)	1.56 (0.94-2.57)	0.084

* *P* < 0.05

**Table 3:** Multivariate Analysis of Factors Associated with Mental Health of PHD

Item number	Variables		PHQ-9 (depressive mood)		GAD-7 (Anxiety)		PSS (perceived stress)		SPS-6 (work-related self-efficacy)	
			AOR(all,glmulti)	*P* value	AOR(all,glmulti)	*P* value	AOR(all,glmulti)	*P* value	AOR(all,glmulti)	*P* value
**Demographic and occupational characteristics**										
Q.1-2	Level of education	College of medicine	NA	NA	NA	NA	NA	NA	NA	NA
		Medical school	NA	NA	NA	NA	NA	NA	NA	NA
		Master's degree	NA	NA	NA	NA	NA	NA	NA	NA
		Doctoral degree	NA	NA	NA	NA	NA	NA	NA	NA
Q.1-3	Marriage	No	NA	NA	NA	NA	NA	NA	1 [reference]	
		Yes	NA	NA	NA	NA	NA	NA	1.62 (0.88-2.97)	0.121
Q.1-4	Children	No	NA	NA	NA	NA	NA	NA	NA	NA
		Yes	NA	NA	NA	NA	NA	NA	NA	NA
Q.1-6	Training	General physician	NA	NA	NA	NA	1 [reference]		NA	NA
		Finished Intern year	NA	NA	NA	NA	0.56 (0.30-1.04)	0.067	NA	NA
		Specialist	NA	NA	NA	NA	0.59 (0.30-1.16)	0.127	NA	NA
Q.1-7	PHD year	First year	NA	NA	not selected		not selected		NA	NA
		Second year	NA	NA	not selected		not selected		NA	NA
		Third year	NA	NA	not selected		not selected		NA	NA
**Working environment in relation to the COVID-19 dispatch**										
Q.2-1	Prior Education	< 8 hours	NA	NA	NA	NA	not selected		NA	NA
		>= 8 hours	NA	NA	NA	NA	not selected		NA	NA
Q.2-2	Notice	< 1 week	not selected		1 [reference]		not selected		not selected	
		>= 1week	not selected		0.49 (0.23-1.04)	0.063	not selected		not selected	
Q.4-1-1	Working hour	< 35 hours/week	1 [reference]		NA	NA	NA	NA	NA	NA
		35-45 hours/week	**2.51 (1.10-6.03)**	0.033*	NA	NA	NA	NA	NA	NA
		> 45 hours/week	**3.24 (1.34-8.20)**	0.01*	NA	NA	NA	NA	NA	NA
Q.4-1-2	Nuber of swabs per day	<10	NA	NA	NA	NA	NA	NA	not selected	
		>=10 & <20	NA	NA	NA	NA	NA	NA	not selected	
		>=20 & <30	NA	NA	NA	NA	NA	NA	not selected	
		>=30 & <40	NA	NA	NA	NA	NA	NA	not selected	
		>=40	NA	NA	NA	NA	NA	NA	not selected	
Q.4-1-4	PPE	insufficient	**1 [reference]**		**1 [reference]**		**1 [reference]**		NA	NA
		sufficient	**0.32 (0.15-0.65)**	0.002*	**0.38 (0.17-0.84)**	0.015*	**0.36 (0.18-0.70)**	0.003*	NA	NA
**Working location of dispatch for COVID-19**										
Q. 2-3	Worked at own area	No	NA	NA	NA	NA	not selected		NA	NA
		Yes	NA	NA	NA	NA	not selected		NA	NA
Q.2-4	Dispatched	No	NA	NA	NA	NA	NA	NA	NA	NA
		Yes	NA	NA	NA	NA	NA	NA	NA	NA
Q.2-7	Dispatch to epicenter	No	NA	NA	NA	NA	NA	NA	NA	NA
		Yes	NA	NA	NA	NA	NA	NA	NA	NA
	Screening center	No	**1 [reference]**		NA	NA	**1 [reference]**		NA	NA
		Yes	**6.07 (1.61-40.50)**	0.022*	NA	NA	**2.90 (1.39-6.48)**	0.006*	NA	NA
	Airport qurantine	No	NA	NA	NA	NA	NA	NA	1 [reference]	
		Yes	NA	NA	NA	NA	NA	NA	1.90 (0.98-3.68)	0.057
	Makeshift shelters	No	NA	NA	NA	NA	NA	NA	NA	NA
		Yes	NA	NA	NA	NA	NA	NA	NA	NA
	Isolation facility	No	NA	NA	NA	NA	NA	NA	NA	NA
		Yes	NA	NA	NA	NA	NA	NA	NA	NA
	Hospital ICU	No	NA	NA	NA	NA	NA	NA	NA	NA
		Yes	NA	NA	NA	NA	NA	NA	NA	NA
**Satisfaction for experience of working at COVID-19 frontline**										
Q.4-2	Subjective workload	NotTough	not selected		1 [reference]		not selected		NA	NA
		Tough	not selected		2.50 (1.01-6.88)	0.058	not selected		NA	NA
Q.4-3	Capability of participating in the decision making	No	not selected		not selected		not selected		not selected	
		Yes	not selected		not selected		not selected		not selected	
Q.4-4	Satisfaction for the monetary compensation	No	NA	NA	NA	NA	**1 [reference]**		NA	NA
		Yes	NA	NA	NA	NA	**0.55 (0.31-0.98)**	0.045*	NA	NA
Q.4-5	Willingness to further volunteering for dispatch	No	not selected		not selected		not selected		**1 [reference]**	
		Yes	not selected		not selected		not selected		**0.47 (0.26-0.82)**	0.009*
Q.4-6	Consulted with psychiatrist	No	NA	NA	NA	NA	NA	NA	NA	NA
		Yes	NA	NA	NA	NA	NA	NA	NA	NA
**Perceived threat of COVID-19 infection**										
Q.3-1	COVID-19 duty puts me at great risk	No	1 [reference]		1 [reference]		1 [reference]		not selected	
		Yes	2.32 (0.95-6.35)	0.079	3.18 (1.09-11.74)	0.051	1.49 (0.87-2.54)	0.148	not selected	
Q.3-2	I feel more stress for COVID-19 duty than other tasks	No	1 [reference]		NA	NA	**1 [reference]**		**1 [reference]**	
		Yes	4.65 (1.18-31.33)	0.054	NA	NA	**2.04 (1.03-4.11)**	0.042*	**4.58 (2.32-9.93)**	<0.001*
Q.3-3	I can accept the risk of caring for COVID-19 patients	No	**1 [reference]**		not selected		NA	NA	NA	NA
		Yes	**0.35 (0.16-0.77)**	0.008*	not selected		NA	NA	NA	NA
Q.3-4	I am afraid of falling ill with COVID-19	No	NA	NA	NA	NA	NA	NA	NA	NA
		Yes	NA	NA	NA	NA	NA	NA	NA	NA
Q.3-5	I have little control over whether I get infected or not	No	not selected		not selected		not selected		not selected	
		Yes	not selected		not selected		not selected		not selected	
Q.3-6	I have little chance of survival if I were to get COVID-19	No	not selected		**1 [reference]**		NA	NA	NA	NA
		Yes	not selected		**8.41 (1.53-48.60)**	0.013*	NA	NA	NA	NA
Q.3-7	If possible, I want to resign from COVID-19 duty	No	not selected		not selected		**1 [reference]**		not selected	
		Yes	not selected		not selected		**2.48 (1.46-4.24)**	0.001*	not selected	
Q.3-8	I am afraid I will pass COVID-19 to others	No	NA	NA	NA	NA	NA	NA	NA	NA
		Yes	NA	NA	NA	NA	NA	NA	NA	NA
Q.3-9	Family & friends are worried they might get infected thru me	No	not selected		**1 [reference]**		not selected		NA	NA
		Yes	not selected		**6.50 (2.05-30.26)**	0.005*	not selected		NA	NA
Q.3-10	People avoid me because of my COVID-19 duty	No	**1 [reference]**		not selected		not selected		NA	NA
		Yes	**2.10 (1.04-4.20)**	0.037*	not selected		not selected		NA	NA

* *P* < 0.05

## Results

### Demographics and working experiences at frontline of COVID-19 for PHDs

Among the 1917 PHDs asked to participate, 350 (18.3%) completed the survey. Table 1 summarizes the demographics and dispatch experiences of all study participants. Participants were males aged 24–34 years. Most were medical school graduates (94%) who had not yet completed their internship training (62.9%), and many were in their first year as PHDs (41.7%). With notification of impending dispatch to the COVID-19 frontline, usually within 7 days of departure (77.1%), they were provided with an average of 3.96 h of education. They were then dispatched mostly to the COVID-19 screening center (85.1%) within (86.6%) or outside (64.9%) their pre-station PHD location, including the Daegu metropolitan area (35.1%; a major outbreak region from February to March of 2020).

At the dispatch location, the PHDs worked an average of 36.6 h per week and performed an average of 19.7 nasopharyngeal swabs per day. Participant ratings were as follows (average points out of 10 possible): subjective workload, 5.7; perceived ability to participate in decision making, 4.2; satisfaction with monetary compensation, 3.5. Furthermore, the score for the intention to volunteer further for the COVID-19 front line was 3.9 points out of 10 (on average). Most were afraid that they would get infected (82.6%) and transfer COVID-19 to someone else (86%). They also felt more distress during COVID-19 frontline duty than during their other duties as a PHD (75.4%). Nonetheless, the PHDs accepted the risk of caring for COVID-19 patients (84.6%).

### Explanatory model of mental health for PHDs: multivariate logistic regression

With regard to self-reported mental health status as of August 2020, of all participating PHDs (*N* = 350), 53 (15.1%) reported a moderate or severe depressive mood (PHQ-9 total score ≥ 10) and were classified in the depressed subgroup [33]. With respect to anxiety, moderate or severe anxiety (GAD-7 scores ≥10) was reported by 40 PHDs (11.4%; anxious subgroup) [34, 39]. Also, 160 PHDs (45.7%) reported higher perceived stress (PSS total score ≥ 18; Figure S1(A)) and were classified as distressed [36]. Finally, 121 PHDs (34.6%) exhibited lowered work-related self-efficacy (SPS-6 total score ≥ 19, Figure S1(B)) or presenteeism [37]. Binary logistic regression analyses were performed using the *glmulti* function in R software to ascertain the effects of the dispatch experience at the COVID-19 frontline on the likelihood that a PHD would suffer depressive mood, anxiety, perceived stress, or lowered self-efficacy at work [40]. Results of the univariate binary logistic regression analyses are demonstrated in Table 2. Also, explanatory variables comprising the best multivariate logistic regression model (with minimum value of Akaike information criterion) in predicting the mental health outcome of PHDs (Table 3) are presented below, with adjusted odds ratios (AORs) and 95% confidence intervals (CIs) [40].

First, higher odds of depressive mood (PHQ-9 total score ≥ 10) were found among those with longer working hours during dispatch [AOR = 2.51 (35–45 h/week, 95% CI = 1.10–6.03, *P* = 0.033) and 3.24 (> 45 h/week, 95% CI = 1.34–8.20, *P* = 0.01)], working at the COVID-19 screening center (AOR = 6.07, 95% CI = 1.61–40.50, *P* = 0.022), and who perceived that people would avoid them because of their COVID-19 duty (AOR = 2.10, 95% CI = 1.04–4.20, *P* = 0.037). By contrast, an adequate supply of protective equipment (AOR = 0.32, 95% CI = 0.15–0.65, *P* = 0.002) and a proactive response to perceived threats, such as “I can accept the risk of caring for COVID-19 patients” (AOR = 0.35, 95% CI = 0.16–0.77, *P* = 0.008), were associated with lower odds of depressive mood.

Second, PHDs with moderate or severe anxiety (GAD-7 total score ≥ 10) reported severe levels of perceived threats, as expressed by “I have little chance of survival if I were to get COVID-19” (AOR = 8.41, 95% CI = 1.53–48.60, *P* = 0.013) and “My family and friends are worried they might get infected with COVID-19 through me” (AOR = 6.50, 95% CI = 2.05–30.26, *P* = 0.005). An adequate supply of protective equipment for COVID-19 duty was related to lower odds of anxiety (AOR = 0.38, 95% CI = 0.17–0.84, *P* = 0.015).

Third, higher perceived stress (PSS total score ≥ 18), including higher perceived threats such as those expressed by “I feel more stress during COVID-19 duty than during other tasks” (AOR = 2.04, 95% CI = 1.03–4.11, *P* = 0.042) and “If it were possible, I would resign from COVID-19 duty” (AOR = 2.48, 95% CI = 1.46–4.24, *P* = 0.001), was associated with assignment to a COVID-19 screening center (AOR = 2.90, 95% CI = 1.39–6.48, *P* = 0.006). By contrast, PHDs who were provided with adequate protective equipment (AOR = 0.36, 95% CI = 0.18–0.70, *P* = 0.003) and who were satisfied with the monetary compensation (AOR = 0.55, 95% CI = 0.31–0.98, *P* = 0.045) were at lower risk of perceived stress. Finally, PHDs with lowered self-efficacy at work or those exhibiting presenteeism (SPS-6 total score ≥ 19) felt more stress during COVID-19 duty compared to other assignments (AOR = 4.58, 95% CI = 2.32–9.93, *P* < 0.001); a willingness to further volunteer for COVID-19 dispatch was associated with lower odds of presenteeism (AOR = 0.47, 95% CI = 0.26–0.82, *P* = 0.009).

## Discussion

### Mental health of PHDs drafted to the frontline of COVID-19

In the current cross-sectional online survey that enrolled 350 PHDs (= 18.3% of population number), perceived distress, lowered self-efficacy at work, anxiety, and depressive mood were reported by 45.7, 34.6, 11.4, and 15.1% of the public health doctors, respectively. This result is in concordance with other studies that demonstrated higher prevalence of mental symptoms in healthcare professionals at frontline of pandemic situation. After working experiences at frontline of COVID-10 pandemic, about 24.7–50.4%, 19.8–44.6%, and 21.9–71.5% of healthcare professionals in China or Italy reported depressive mood, anxiety, and perceived distress, which were higher prevalence compared to those at baseline (=prior to the working experiences at frontline of COVID-19 pandemic) [41–44]. In addition, lowered self-efficacy at work (or a lower sense of personal accomplishment) was also reported from 21.4–22.7% of healthcare professionals of Libya during COVID-19 pandemic [45, 46]. For the cases of MERS outbreak, 26.6% of doctors who dealt with the MERS outbreak in South Korea [38] and 27.5% of doctors responding to the SARS outbreak in Taiwan exhibited depression [47]. During the SARS outbreak, 89% of healthcare workers who were in high-risk situations in Hong Kong reported psychological symptoms [48]. Specific viral characteristics of COVID-19, e.g., its high infectivity and often-fatal outcome in older populations, might have played a role in the experiences and feelings of healthcare workers (including PHDs). Although similarities between these cases and PHDs are evident, some characteristics unique to PHDs and their working conditions should be considered. First, PHDs are younger-aged (20s–30s) male doctors who are enrolled in mandatory military service. The average age of the study population was 28.9 years (SD = 2.11 years). Second, the intrinsic nature of the PHD system, i.e., a non-voluntary working force who must follow government orders and are stationed far from home, could have affected the psychology of these frontline physicians in a different way from other healthcare workers. These circumstances are unique and should be documented.

### Personal protective equipment, Workhours at screening center, and mental health

Multivariate logistic regression demonstrated that insufficient supply of personal protective equipment during the working experiences at frontline of COVID-19 pandemic might be a predictor of depressive mood, anxiety, and distress for PHDs. In addition, draft to the COVID-19 frontline of screening center and longer weekly workhours were also included as predictors in the best multivariate binary logistic regression model of depressive mood for PHDs. The current study result is in concordance with recent studies of healthcare workers in German or Italy during COVID-19 that showed associations between working at COVID-19 frontline versus higher level of perceived stress or post-traumatic symptoms [44, 49]. Further, other studies that examined mental health of medical professionals at frontline of COVID-19 pandemic also demonstrated increased odds of mental health worsening (depressive mood, anxiety, or burnout) in presence of working environments such as insufficient supply of personal protective equipment [50–54], endorsed barriers at working [55], increased physical and psychological workload [51, 56, 57], reduced amount of day-off and successive period of active duty after 24-h shifts [50]. Collectively, sufficient and timely supply of personal protective equipment and efficient distribution of rest period between the active duty are required for protection of mental health for PHDs at frontline of pandemic.

### Perceived threat of infection, stigma and social rejection, and mental health

In addition, psychosocial aspects of working experience at frontline of COVID-19 also significantly predicted mental health of PHDs. First, perceived threat of infection and fear of death comprised the best multivariate binary logistic regression model of predicting anxiety of PHDs. These results are in accordance with cases of medical healthcare workers that demonstrated positive associations between the severity of perceived threat for COVID-19 or being at risk of contact with COVID-19 patients versus reports of depressive symptoms, anxiety, and insomnia [44, 58, 59]. Fear of contracting COVID-19 at work is associated with higher emotional exhaustion and depersonalization [45] and could be one of the most prominent distress for medical professionals with frequent contacts with COVID-positive patients [54, 57, 60]. Second, perceived stigma from family and friends (worries for possible transmission of infection through PHDs at frontline) and rejection from neighborhood [ex. local residents forcefully entered a PHD’s house and sprayed disinfectants as the PHD had just finished duty at frontline of pandemic and returned to his original workplace as PHD (https://www.seoul.co.kr/ news/newsView.php?id=20200319500158&wlog_tag3=naver)] predicted anxiety and depressive mood of PHDs, respectively. Another study of healthcare workers in Libya also showed significant associations between the stigmatization and depressive symptoms and anxiety [61]. Therefore, greater psychosocial support from family, friends, supervisors and better cooperation between colleagues at workplace are important [15, 57]. Also, proper educational training on COVID-19 to the healthcare professionals in addition to the clearer dissemination of disease-related information to the general population could help to reduce anxiety in PHDs on the frontline of COVID-19 pandemic [15, 18].

### Needs of psychosocial support for protecting mental health of PHD

Protection of mental health for healthcare professionals at frontline of pandemic situation is critical, not only for successful termination of COVID-19 pandemic in the long term [62] but also for prevention of suffer of PHDs and military medical staffs from post-traumatic stress symptoms after deployment [63, 64]. Also, the current study revealed possible protective factors of mental health for PHDs with working experiences at frontline of COVID-19 pandemic. First, satisfaction for monetary compensation was associated with lower level of distress. This is in line with another recent study that demonstrated positive association between the monetary compensation and willingness of nurses to care for patients with COVID-19 [65]. Therefore, application of the remuneration system for PHDs that better reflects the risks of working environments [65] would be more beneficial in enhancing the willingness of PHDs to volunteer duty at frontline of pandemic. Second, proactive coping strategy of altruistic acceptance for managing infected patients at frontline and willingness to volunteer further duty at frontline were predictive of less depressive mood and higher work-related self-efficacy, respectively. As a matter of fact, prolonged exposure to the working environment of pandemic frontline could result in biological injury (of COVID-19 infection) and psychological injury (of mental health worsening) of PHDs [66]. A recent study of medical professionals in Wuhan of China emphasizes the importance of timely mental health supports for frontline workers during pandemic [43]. Psychological supports [67] that provide strategies of positive coping (altruistic acceptance of work-related risks, motivation to learning different skills, and humor) [15, 67], psychological debriefing [15, 68], and regular monitoring of mental health [67] aiming to delivering proper treatment by psychiatrists would be effective in protecting mental health of PHDs at frontline. In addition, short-term and long-term plans to support mental health of healthcare workers during and after the COVID-19 pandemic is warranted [54].

### Limitations

The current study has some limitations to be addressed. First, some possible associated factors of the working experiences at pandemic frontline, such as experience of quarantine during this pandemic and perceived social supports from family/supervisors and colleagues/neighbors [15] were not covered in this study. Second, duration of data collection (August 2021) was relatively short. Third, approximately half of the participants were still actively working in the COVID-19 field at the time of the survey, with other half already had moved from the frontline of COVID-19 at time-point of study participation.

## Conclusions

To the best of the authors’ knowledge, the current study is first to examine the status and possible associated factors of mental health among young medical doctors drafted for COVID-19 frontline duty. For better mental health of healthcare professionals at frontline of pandemic including PHDs, sufficient supply of personal protective equipment and training of how to prevent infection at frontline, proper workhours and satisfactory monetary compensation, and psychological care programs are required.

## Supplementary Information

**Additional file 1: S1.** English version of online survey. **S2.** R code for multivariate logistic regression. **Figure S1.** (A) Distribution of PSS (perceived stress scale) total score (B) Distribution of total score for COVID-19 version of SPS-6 (Stanford Presenteeism Scale-6) (M = 350).

## Data Availability

The datasets used and/or analyzed during the study are available from the corresponding author on reasonable request.
