# Supplementary material for: Associations between the working experiences at frontline of COVID-19 pandemic and mental health of Korean public health doctors
Source: BMC Psychiatry. 2021 Jun 9;21:298. doi: 10.1186/s12888-021-03291-2 (PMC8188159; doi:10.1186/s12888-021-03291-2)
Supplement: Supplementary file 1 — Additional file 1: S1. English version of online survey. S2. R code for multivariate logistic regression. Figure S1. (A) Distribution of PSS (perceived stress scale) total score (B) Distribution of total score for COVID-19 version of SPS-6 (Stanford Presenteeism Scale-6) (M = 350). [file 12888_2021_3291_MOESM1_ESM.docx]

Associations between the working experiences at frontline of COVID-19 pandemic and mental health of Korean public health doctors

Sangyoon Han, MD^1*^; Sejin Choi, MD^2,5*^; Seung Hyun Cho, MD^3^; Joonhyuk Lee, MD^4^; Je-Yeon Yun, MD, PhD^5,6^

* These authors contributed equally to this work

^1^Eastern Seoul Detention Center, Ministry of Justice, Republic of Korea

^2^Seoul Detention Center, Ministry of Justice, Republic of Korea

^3^Gaedo Public Health Center, Yeosu, Republic of Korea

^4^Hajang Public Health Center, Samcheok, Republic of Korea

^5^Seoul National University Hospital, Seoul, Republic of Korea

^6^Yeongeon Student Support Center, Seoul National University College of Medicine, Seoul, Republic of Korea

**Corresponding Author:** Je-Yeon Yun, MD, PhD, Seoul National University Hospital, 101, Daehang-ro, Jongno-gu, Seoul 03080, Republic of Korea ([tina177@snu.ac.kr](mailto:tina177@snu.ac.kr)).

**S1. English version of online survey**

**1. This section asks about your demographics and experience as a practitioner.**

1-1. Please respond your age.

1-2. What is your final degree?

l  College of Medicine (Bachelor of Medicine)

l  Medical School (Master of Medicine)

l  Master’s Degree

l  Doctoral Degree

1-3. What is your status marriage?

l  Married

l  Not Married

1-4. Do you have any children currently under your care?

l  Yes

l  No

1-5. Before undertaking COVID-19 duties how many months of clinical experience do you possess? (months)

1-6. Please select your level of training as physician.

l  General Practitioner (No training after acquiring M.D.)

l  Intern (First year of training)

l  Finished Residency and acquired a specialty

1-7. Please select your year of PHD duty.

l  First year

l  Second year

l  Third year

**2. This section asks you about the working environment of COVID-19 duty.**

2-1. How many hours of quarantine education did you receive before being put to work? (hours)

2-2. On average, how many days of notice did you get before being summoned to a certain COVID-19 duty? (days, if only hours respond 0)

2-3. Do you have any experience of working at a COVID-19 triage center of your stationed city or county?

l  Yes

l  No

2-4. Have you been dispatched to a facility that was not under the jurisdiction of your pre-stationed city or county?

l  Yes

l  No

2-5-1. What is the starting date of your very first COVID-19 duty? ** If you have been on several duties, please state the starting date of your very first one

2-5-2. What is the date of your last COVID-19 duty? ** If you have been on several duties, please state the last day of your final one. If you are currently on duty, please respond today’s date.

2-6. Please select all the regions you have been placed in during your COVID-19 duties.  ** [Include your pre-stationed region if you have an experience of COVID-19 duty in that region] Select all.

n  Seoul

n  Busan

n  Daegu

n  Incheon

n  Gwangju

n  Daejeon

n  Ulsan

n  Sejong

n  Gyeonggido

n  Gangwondo

n  Chungchungbukdo

n  Chungchungnamdo

n  Jeollabukdo

n  Jeollanamdo

n  Gyeonsangbukdo

n  Gyeonsangnamdo

n  Jeju

2-7. Select all the facilities you have an experience of performing COVID-19 duties.  ** [Include your experience at facilities within your pre-stationed region] Select all.

n  Triage center of public health centers

n  Triage center of quarantine facility

n  Saenghwal Chiryo Center (Facility for COVID-19 positives with mild condition)

n  Imsi Saenghwal Sookso (Temporary quarantine facility for new visitors to the country)

n  Hospital

**3. This section is about feeling of threat that you might have experienced during COVID-19 duties. Please recall the feeling that you had when you were on duty. (modified from "Perception of threat of MERS" in Um et al. (2017)**

3-1. COVID-19 duty puts me at great risk.

l  Never

l  Not so much

l  Unsure

l  Possibly

l  Certainly

3-2. I feel more stress during COVID-19 duty than before.

l  Never

l  Not so much

l  Unsure

l  Possibly

l  Certainly

3-3. I can accept the risk of caring for COVID-19 patients.

l  Never

l  Not so much

l  Unsure

l  Possibly

l  Certainly

3-4. I am afraid of falling ill with COVID-19

l  Never

l  Not so much

l  Unsure

l  Possibly

l  Certainly

3-5. I have little control over whether I get infected or not.

l  Never

l  Not so much

l  Unsure

l  Possibly

l  Certainly

3-6. I have little chance of survival if I were to get COVID-19

l  Never

l  Not so much

l  Unsure

l  Possibly

l  Certainly

3-7. If it were possible, I want to resign from COVID-19 duty.

l  Never

l  Not so much

l  Unsure

l  Possibly

l  Certainly

3-8. I am afraid I will pass COVID-19 to others.

l  Never

l  Not so much

l  Unsure

l  Possibly

l  Certainly

3-9. My family and friends are worried they might get infected through me.

l  Never

l  Not so much

l  Unsure

l  Possibly

l  Certainly

3-10. People avoid me because of my COVID-19 duty

l  Never

l  Not so much

l  Unsure

l  Possibly

l  Certainly

**4. This section asks about your experience during the COVID-19 duties.**

4-1-1. What was the average work hour of your COVID-19 duties per day? (In hours)

4-1-2. On average, how many COVID-19 samples did you take in a day?

4-1-3. On average, how much rest time (including lunch hour) did you get per day while in COVID-19 duty ?

4-1-4. Were you provided with enough protective equipment while on your COVID -19 duty?

l  Always adequate

l  Sometimes inadequate

l  Often inadequate

l  Always inadequate

l  Never provided

4-2. What was your subjective feeling of workload during your COVID-19 duty. (Out of score 10)

l  0 (Never felt hard)

l  1

l  2

l  3

l  4

l  5

l  6

l  7

l  8

l  9

l  10 (Hardest work ever done)

4-3. Did you feel your opinion about work or working environment was aptly considered and applied well during your COVID-19 duties? (Out of score of 10)

l  0 (Never applied)

l  1

l  2

l  3

l  4

l  5

l  6

l  7

l  8

l  9

l  10(Applied to satisfaction)

4-4. How much were you satisfied with the compensations of COVID-19 duties? (Out of score of 10)

l  0(Not at all satisfied)

l  1

l  2

l  3

l  4

l  5

l  6

l  7

l  8

l  9

l  10(Completely satisfied)

4-5. If you had the choice to participate in COVID-19 duties how much will you be willing to volunteer? (Out of score of 10)

l  0(Will never volunteer)

l  1

l  2

l  3

l  4

l  5

l  6

l  7

l  8

l  9

l  10(Will absolutely volunteer)

4-6. Do you have any experience of consulting with a psychiatrist qt any time after or during?

l  Yes

l  No

**5. Over the last two weeks, how often have you been bothered by any of the following problems?**

5-1. Little interest or pleasure in doing things?

l  Not at all

l  2-6 days

l  7-12 days

l  Nearly every day

5-2. Feeling down, depressed, or hopeless?

l  Not at all

l  2-6 days

l  7-12 days

l  Nearly every day

5-3. Trouble falling or staying asleep, or sleeping too much?

l  Not at all

l  2-6 days

l  7-12 days

l  Nearly every day

5-4. Poor appetite or overeating?

l  Not at all

l  2-6 days

l  7-12 days

l  Nearly every day

5-5. Moving or speaking so slowly that other people could have noticed?
Or the opposite - being so fidgety or restless that you have been moving around a lot more than usual?

l  Not at all

l  2-6 days

l  7-12 days

l  Nearly every day

5-6. Feeling tired or having little energy?

l  Not at all

l  2-6 days

l  7-12 days

l  Nearly every day

5-7. Feeling bad about yourself - or that you are a failure or have let yourself or your family down?

l  Not at all

l  2-6 days

l  7-12 days

l  Nearly every day

5-8. Trouble concentrating on things, such as reading the newspaper or watching television?

l  Not at all

l  2-6 days

l  7-12 days

l  Nearly every day

5-9. Thoughts that you would be better off dead, or of hurting yourself in some way?

l  Not at all

l  2-6 days

l  7-12 days

l  Nearly every day

**6. Over the last 2 weeks, how often have you been bothered by the following problems?**

6-1. Feeling nervous, anxious, or on edge

l  Not at all sure

l  Several Days

l  Over half the days

l  Nearly every day

6-2. Not being able to stop or control worrying

l  Not at all sure

l  Several Days

l  Over half the days

l  Nearly every day

6-3. Worrying too much about different things

l  Not at all sure

l  Several Days

l  Over half the days

l  Nearly every day

6-4. Trouble relaxing

l  Not at all sure

l  Several Days

l  Over half the days

l  Nearly every day

6-5. Being so restless that it's hard to sit still

l  Not at all sure

l  Several Days

l  Over half the days

l  Nearly every day

6-6. Becoming easily annoyed or irritable

l  Not at all sure

l  Several Days

l  Over half the days

l  Nearly every day

6-7. Feeling afraid as if something awful might happen

l  Not at all sure

l  Several Days

l  Over half the days

l  Nearly every day

**7. The questions in this scale ask you about your feelings and thoughts during the last month. In each case, you will be asked to indicate by circling how often you felt or thought a certain way.**

7-1. In the last month, how often have you been upset because of something that happened unexpectedly?

l  Never

l  Almost Never

l  Sometimes

l  Fairly Often

l  Very Often

7-2. In the last month, how often have you felt that you were unable to control the important things in your life?

l  Never

l  Almost Never

l  Sometimes

l  Fairly Often

l  Very Often

7-3. In the last month, how often have you felt nervous and “stressed”?

l  Never

l  Almost Never

l  Sometimes

l  Fairly Often

l  Very Often

7-4. In the last month, how often have you felt confident about your ability to handle your personal problems?

l  Never

l  Almost Never

l  Sometimes

l  Fairly Often

l  Very Often

7-5. In the last month, how often have you felt that things were going your way?

l  Never

l  Almost Never

l  Sometimes

l  Fairly Often

l  Very Often

7-6. In the last month, how often have you found that you could not cope with all the things that you had to do?

l  Never

l  Almost Never

l  Sometimes

l  Fairly Often

l  Very Often

7-7. In the last month, how often have you been able to control irritations in your life?

l  Never

l  Almost Never

l  Sometimes

l  Fairly Often

l  Very Often

7-8. In the last month, how often have you felt that you were on top of things?

l  Never

l  Almost Never

l  Sometimes

l  Fairly Often

l  Very Often

7-9. In the last month, how often have you been angered because of things that were outside of your control?

l  Never

l  Almost Never

l  Sometimes

l  Fairly Often

l  Very Often

7-10. In the last month, how often have you felt difficulties were piling up so high that you could not overcome them?

l  Never

l  Almost Never

l  Sometimes

l  Fairly Often

l  Very Often

**8. Following questions will ask about your working efficiency as a public health doctor in the last 4 weeks. Please respond regarding what has changed after COVID-19 duty.**

8-1. Because of my experience in COVID-19 duty, the stresses of my job were much harder to handle..

l  Strongly disagree

l  Somewhat disagree

l  Uncertain

l  Somewhat agree

l  Strongly agree

8-2. Despite having my experience in COVID-19 duty, I was able to finish hard tasks in my work.

l  Strongly disagree

l  Somewhat disagree

l  Uncertain

l  Somewhat agree

l  Strongly agree

8-3. My experience in COVID-19 duty distracted me from taking pleasure in my work.

l  Strongly disagree

l  Somewhat disagree

l  Uncertain

l  Somewhat agree

l  Strongly agree

8-4. I felt hopeless about finishing certain work tasks, due to my experience in COVID-19 duty.

l  Strongly disagree

l  Somewhat disagree

l  Uncertain

l  Somewhat agree

l  Strongly agree

8-5. At work, I was able to focus on achieving my goals despite my experience in COVID-19 duty.

l  Strongly disagree

l  Somewhat disagree

l  Uncertain

l  Somewhat agree

l  Strongly agree

8-6. Despite having my experience in COVID-19 duty, I felt energetic enough to complete all my work.

l  Strongly disagree

l  Somewhat disagree

l  Uncertain

l  Somewhat agree

l  Strongly agree

**S2. R code for multivariate logistic regression**

glmulti.logistic.out <-

glmulti(PHQ2or3 ~ Notice_group_binary+Working_hour_category+PPE_binary+Screening_binary+MERS1_binary+MERS2_binary+MERS3_binary+MERS5_binary+MERS6_binary+MERS7_binary+MERS9_binary+MERS10_binary+T_group+O_group+V_group,

data = D,

level = 1, # No interaction considered

method = "h", # Exhaustive approach

crit = "aic", # AIC as criteria

confsetsize = 5, # Keep 5 best models

plotty = F, report = F, # No plot or interim reports

fitfunction = "glm",

family=binomial) # glm function

glmulti.logistic.out@formulas

summary(glmulti.logistic.out@objects[[1]])

glmulti.logistic.out <-

glmulti(GAD2or3 ~ PHD_year+Notice_group_binary+PPE_binary+

MERS1_binary+MERS3_binary+MERS5_binary+MERS6_binary

+MERS7_binary+MERS9_binary+MERS10_binary+

T_group+O_group+V_group,

data = D,

level = 1, # No interaction considered

method = "h", # Exhaustive approach

crit = "aic", # AIC as criteria

confsetsize = 5, # Keep 5 best models

plotty = F, report = F, # No plot or interim reports

fitfunction = "glm",

family=binomial) # glm function

glmulti.logistic.out@formulas

summary(glmulti.logistic.out@objects[[1]])

glmulti.logistic.out <-

glmulti(PSS_group ~Training+PHD_year+Prev_edu_1d+Notice_group_binary+PPE_binary+Self_area+Screening_binary+MERS1_binary+MERS2_binary+MERS5_binary+MERS7_binary+MERS9_binary+MERS10_binary+T_group+O_group+P_group+V_group,

data = D,

level = 1, # No interaction considered

method = "h", # Exhaustive approach

crit = "aic", # AIC as criteria

confsetsize = 5, # Keep 5 best models

plotty = F, report = F, # No plot or interim reports

fitfunction = "glm",

family=binomial) # glm function

glmulti.logistic.out@formulas

summary(glmulti.logistic.out@objects[[2]])

glmulti.logistic.out <-

glmulti(SPS_group ~ Marriage+Notice_group_binary+Quarantine_binary+Swab_category+MERS1_binary+MERS2_binary+MERS5_binary+MERS7_binary+O_group+V_group,

data = D,

level = 1, # No interaction considered

method = "h", # Exhaustive approach

crit = "aic", # AIC as criteria

confsetsize = 5, # Keep 5 best models

plotty = F, report = F, # No plot or interim reports

fitfunction = "glm",

family=binomial) # glm function

glmulti.logistic.out@formulas

summary(glmulti.logistic.out@objects[[2]])

**Figure S1.** (A) Distribution of PSS (perceived stress scale) total score (B) Distribution of total score for COVID-19 version of SPS-6 (Stanford Presenteeism Scale-6) (M = 350)

| 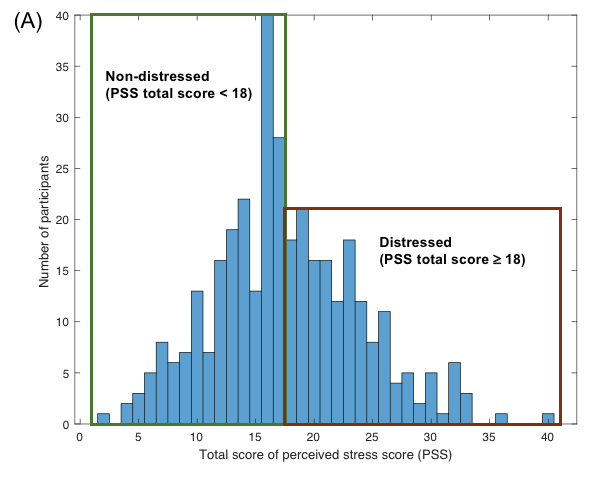 | 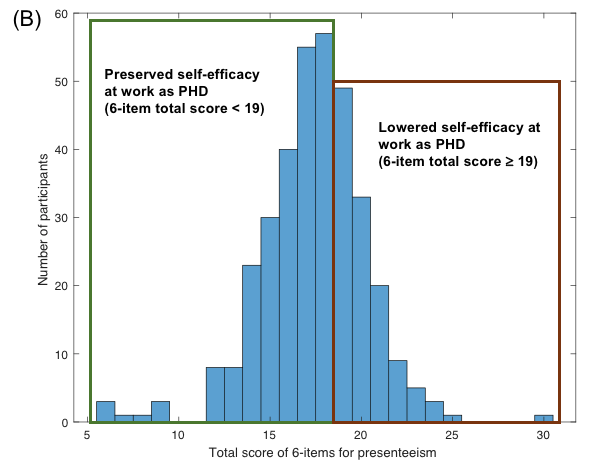 |
| --- | --- |

±
